# Novel Process of Intrathymic Tumor-Immune Tolerance through CCR2-Mediated Recruitment of Sirpα^+^ Dendritic Cells: A Murine Model

**DOI:** 10.1371/journal.pone.0041154

**Published:** 2012-07-16

**Authors:** Tomohisa Baba, Mohamed El Sherif Badr, Utano Tomaru, Akihiro Ishizu, Naofumi Mukaida

**Affiliations:** 1 Division of Molecular Bioregulation, Cancer Research Institute, Kanazawa University, Kanazawa, Ishikawa, Japan; 2 Department of Pathology/Pathophysiology, Graduate School of Medicine, Hokkaido University, Sapporo, Hokkaido, Japan; 3 Faculty of Health Sciences, Hokkaido University, Sapporo, Hokkaido, Japan; Otto-von-Guericke University Magdeburg, Germany

## Abstract

Immune surveillance system can detect more efficiently secretory tumor-specific antigens, which are superior as a target for cancer immunotherapy. On the contrary, immune tolerance can be induced in the thymus when a tumor antigen is massively secreted into circulation. Thus, the secretion of tumor-specific antigen may have contradictory roles in tumor immunity in a context-dependent manner. However, it remains elusive on the precise cellular mechanism of intrathymic immune tolerance against tumor antigens. We previously demonstrated that a minor thymic conventional dendritic cell (cDC) subset, CD8α^−^Sirpα^+^ cDCs, but not the major subset, CD8α^+^Sirpα^−^ cDCs can selectively capture blood-borne antigens and crucially contribute to the self-tolerance. In the present study, we further demonstrated that Sirpα^+^ cDCs can capture a blood-borne antigen leaking inside the interlobular vascular-rich regions (IVRs). Blood-borne antigen selectively captured by Sirpα^+^ cDCs can induce antigen-specific Treg generation or negative selection, depending on the immunogenicity of the presented antigen. Furthermore, CCR2 expression by thymic Sirpα^+^ cDCs and abundant expression of its ligands, particularly, CCL2 by tumor-bearing mice prompted us to examine the function of thymic Sirpα^+^ cDCs in tumor-bearing mice. Interestingly, tumor-bearing mice deposited CCL2 inside IVRs in the thymus. Moreover, tumor formation induced the accumulation of Sirpα^+^ cDCs in IVRs under the control of CCR2-CCL2 axis and enhanced their capacity to take up antigens, resulting in the shift from Treg differentiation to negative selection. Finally, intrathymic negative selection similarly ensued in CCR2-competent mice once the tumor-specific antigen was secreted into bloodstream. Thus, we demonstrated that thymic Sirpα^+^ cDCs crucially contribute to this novel process of intrathymic tumor immune tolerance.

## Introduction

Immune surveillance system can detect more efficiently secretory tumor-specific antigens than intracellular ones. Thus, it is widely believed that secretory antigens are superior as a target for cancer immunotherapy to intracellular antigens [1], [2]. On the contrary, immune tolerance can be induced when a tumor antigen is massively secreted into circulation. Supporting the latter notion, tumor antigen-specific CD4^+^ T cells were eliminated in the thymus and draining lymph nodes in the mice bearing a tumor, which constitutively secreted an antigen into the circulation [3], [4]. Thus, a secretory tumor antigen may have contradictory roles in tumor immunity in a context-dependent manner. However, it remains elusive on the precise cellular mechanism and molecular regulation of immune tolerance against secretory tumor antigens.

Intrathymic educational processes consist of the induction of negative selection and natural Treg differentiation, and participate in orchestrating central immune tolerance, thereby reducing the risk of developing autoimmune disorders. Negative selection, a cardinal recessive tolerance process, is crucial for the maintenance of immune homeostasis while Treg-mediated immune regulation is a main gateway to dominant tolerance and can intervene in ongoing abnormal immune responses. Central tolerance is accurately executed by several antigen presenting cells (APCs) including medullary thymic epithelial cells (mTECs) and thymic DCs. A transcription factor, autoimmune regulator (AIRE) [5], is selectively expressed in mTECs and to a lower extent, thymic DCs, and constitutively regulates the transcription of tissue-restricted antigens in mTECs, thereby inducing central tolerance, negative selection and Treg differentiation from thymocytes which can recognize the antigens expressed in mTECs [5], [6], [7], [8], [9]. Additionally, some DCs can convey the peripheral self-antigens to the thymus, resulting in the induction of negative selection and Treg differentiation in the DC-dependent manner [10], [11].

Recently, Wu and Shortman classified thymic DCs into three subsets, CD11c^+^CD11b^−^CD8α^+^Sirpα^−^ and CD11c^+^CD11b^+^CD8α^−^Sirpα^+^ cDCs, and CD11c^+^B220^+^ plasmacytoid DCs [12]. Among these three DC subsets, the most abundant CD8α^+^Sirpα^−^ cDCs subset is clustered in the medulla with a low, but effective AIRE expression, and can present endogenous self-antigens. Moreover, they can take up self-antigens from mTECs and cross-present them to the thymocytes [12], [13]. However, it still remains elusive on the intrathymic location and function of the other minor cDC, CD11c^+^CD11b^+^CD8α^−^Sirpα^+^ subset. We previously demonstrated that most Sirpα^+^ cDCs, as opposed to Sirpα^−^ cDCs, are disseminated in close proximity to small vessels in the cortex and inside the perivascular regions [14]. Their unique intrathymic localization allows thymic Sirpα^+^ cDCs to selectively capture intravenously injected antigens across the blood-thymus barrier [14]. Interestingly, mice deficient in a CC chemokine receptor, CCR2, exhibit a selective decrease in the Sirpα^+^ cDC subset in the thymus, with an accumulation of T cells displaying reactivity against serum self-antigen in the periphery [14], suggesting the crucial contribution to the central tolerance against blood-borne antigens.

In the present study, more detailed histological analysis revealed that thymic Sirpα^+^ cDCs were disseminated in the cortex and inside the interlobular vascular-rich regions (IVRs), encompassed with the cortical parenchyma. Moreover, Sirpα^+^ cDCs efficiently captured blood-borne antigen leaking inside the IVRs in a CCR2-dependent manner. Intravenously injected untreated ovalbumin (OVA) protein and highly immunogenic heat-aggregated OVA protein activated thymic Sirpα^+^ cDCs to initiate antigen-specific Treg differentiation and negative selection for OVA-specific TCR transgenic thymocytes, respectively. Moreover, when developed subcutaneously, tumor induced the deposition of a CCR2 ligand, CCL2 inside IVRs, resulting in the enhancement of antigen uptake by intrathymic Sirpα^+^ cDCs. We finally proved that the negative selection rather than Treg differentiation ensued in a CCR2-dependent manner once the tumor-specific antigen was secreted into bloodstream.

## Methods

### Tissues

Human tissues were obtained from autopsy with a written informed consent in accordance with the Declaration of Helsinki. All human studies were approved by the Medical Ethics Committee of Hokkaido University Graduate School of Medicine. Thymic tissues were flash-frozen in liquid nitrogen and stored at −80°C.

### Plasmids

pMYs-IRES-GFP and pLEGFP-N1 retrovirus vectors were purchased from Cell Biolabs and Clontech, respectively. An antagonistic form of CCL2 (7ND) is the amino terminus deleted form of human CCL2 and can exhibit antagonistic activities against CCR2 [15]. 7ND-expressing vector was prepared by cloning to the pMYs-IRES-GFP vector, the human CCL2 NH_2_-terminus–deleted cDNA linked with an epitope FLAG tag in the carboxyl terminal portion [16]. OVA cDNA with a truncated stop codon was amplified by PCR from chicken OVA cDNA and the signal peptide sequence of mouse albumin was synthesized as two complementary oligonucleotides (Gene Design). OVA cDNA linked with and without albumin signal peptide sequence were inserted into pLEGFP-N1 vector, to generate sOVA-GFP and OVA-GFP, respectively.

### Transfectants

The Phoenix packaging cell line was transfected with 7ND, OVA-GFP, or sOVA-GFP retrovirus vector to produce the retrovirus in the culture supernatant, which was subjected to the infection. A murine colorectal cancer cell line, Col26 [17] was infected with the resultant retrovirus to generate clones expressing stably 7ND, OVA-GFP, or sOVA-GFP. The concentration of OVA protein in the culture supernatant (1×10^5^ cells/ml) was determined using Chicken Egg Ovalbumin ELISA kit (Alpha Diagnostic).

### Mice

Specific pathogen-free male BALB/c and C57BL/6 mice were purchased from Charles River Japan, and designated as WT mice. CCR2^−/−^ mice [18] were kindly provided by Dr. William Kuziel (University of Texas San Antonio), and were backcrossed to BALB/c mice for more than 8 generations. CCR2^−/−^ backcrossed to C57BL/6 mice were provided also by Dr. Kuziel via Dr. Motohiro Takeya (Kumamoto University). DO11.10 mice expressing a transgenic TCR that recognizes the OVA_323–339_ peptide in the context of I-A^d^ were kindly provided by Dr. Yasunari Nakamoto (Kanazawa University) and were maintained as heterozygotes. OT-I and OT-II mice express transgenic TCRs that recognize the OVA_257–264_ peptide in the context of H-2K^b^ and OVA_323–339_ peptide in the context of I-A^b^, respectively. These mice were supplied from CARD, Kumamoto University. DO11.10 mice were mated with CCR2^−/−^ mice to generate DO11.10/CCR2^−/−^ mice on a BALB/c background. All animal experiments were approved and performed according to the Guideline for the Care and Use of Laboratory Animals of Kanazawa University (permission number AP-111852).

### Antibodies (Abs)

The following rat anti-mouse mAbs were used; anti-CD4 (RM4-5; BD Biosciences), anti-CD8 (53–6.7; BD Biosciences), anti-CD11b (M1/70; eBioscience), anti-CD25 (PC61; BD Biosciences), anti-CD172a/Sirpα (P84; BD Biosciences), anti-DC-SIGN (5H10; eBioscience), anti-DO11.10 clonotypic TCR (KJ1-26; BD Biosciences), anti-Foxp3 (FJK-16s; eBioscience and MF23; BD Biosciences), and anti-Ly-51 (6C3; Biolegend). Mouse anti-mouse I-A^d^ (AMS-32.1; BD Biosciences), and hamster anti-mouse CD11c (HL-3; BD Biosciences), and anti-mouse CCL2 (2H5; eBioscience) mAbs were used. Rabbit anti-mouse type IV collagen (Col IV) polyclonal Ab was purchased from LSL. Mouse anti-human CD172a/Sirpα (15–414; AbD Serotec) and anti-DC-SIGN (DCN47.5; Miltenyi Biotec) mAbs were used. Isotype-matched control IgGs for individual mouse, rat, and hamster mAbs were purchased from BD Biosciences. Rabbit IgG (Sigma-Aldrich) served as negative controls.

### Cell preparation

Thymus and spleen were obtained from 6- to 7-week old mice. Total thymocytes were isolated by mechanical digestion from thymus. In some experiments, thymus was digested with 0.6 mg/ml collagenase type IV (Sigma-Aldrich) and 25 Kunitz units/ml DNase I (Sigma-Aldrich) at 37°C for 20 min. The resultant single cell suspensions were further separated by density gradient centrifugation using Histopaque-1077 reagent (Sigma-Aldrich). The cells in the interphase were obtained and were used as thymic low-density cells. Bone marrow cells were flushed out with cold RPMI 1640 medium (Sigma-Aldrich) from the femoral and tibial bones.

### RT-PCR

Total RNAs were extracted from tissues using a RNeasy Mini Kit (Qiagen) and then reverse-transcribed using SuperScript III First-Strand Synthesis System (Invitrogen). PCR was done using the cDNAs, 2.5 mM dNTP mix (Takara), TaqDNA polymerase (Takara), and the specific primer sets for *GAPDH* gene (sense: 5′-CAC TGA GCA TCT CCC TCA CA-3′; antisense: 5′-TGG GTG CAG CGA ACT TTA TT-3′), *DC-SIGN* gene (sense: 5′-CAG TTG AAG GCT GGC GTA G-3′; antisense: 5′-ACA AGT TGA GCC CCC ACA T-3′), *SIGNR1* gene (sense: 5′-CAT GCA GGC GAA GAT CAC T-3′; antisense: 5′-TAC CGG GAA GCT GGA GAT C-3′), *SIGNR2* gene (sense: 5′-CTA CTC CCA CGG ACA AGA ACA-3′; antisense: 5′-CCA GTT CTG TTG GAA CTT GGA-3′), *SIGNR3* gene (sense: 5′-GCT GCT TTC CTT CCT GTT CTT-3′; antisense: 5′-TCA GCC TTC AGT TGC ATG AG-3′), and *SIGNR4* gene (sense: 5′-CAG TGG GAA CAC ACA AAG CA-3′; antisense: 5′-CAT TTC TTT GCA GGC AGT CA-3′) for 28 cycles (for GAPDH) or 32 cycles (for others) of 95°C for 30 s, 55°C for 30 s, and 72°C for 30 s.

### Flow cytometry (FCM)

Total thymocytes and thymic low-density cells were stained with various combinations of fluorescent dye-conjugated or non-conjugated specific Abs in magnetic activated cell separation (MACS) buffer (PBS supplemented with 2 mM EDTA and 3% FBS). For non-conjugated Abs, fluorescent-conjugated secondary Abs were used. Intranuclear Foxp3 was stained with the help of FITC anti-mouse Foxp3 staining set (eBioscience). Expression of each molecule was analyzed using FACSCalibur or FACSCanto II (BD Biosciences) with CellQuest Pro software (BD Biosciences) and FlowJo software (TreeStar).

### Immunofluorescence analysis

Six or 15 µm-thick cryostat sections were fixed with cold acetone for 3 min or with 4% paraformaldehyde for 15 min and permeabilized with 0.1% Triton X for 15 min, and incubated with Protein Block Reagent (DAKO) to block non-specific binding. Then, fluorescent immunostaining was done by the standard method. After being washed with 0.05% Tween 20-PBS, slides were mounted in fluorescent mounting medium (VECTOR) with or without DAPI (DAKO). Immunofluorescence was detected in the setting that excluded the non-specific signal of the isotype control, using a fluorescence microscope, BX50 (Olympus), or a confocal laser scanning microscope, LSM510 (Carl Zeiss). DP Controller software (Olympus) and Zen 2007 software (Carl Zeiss) were used for image processing.

### Uptake of antigens by thymic DCs

Alexa Fluor 488- (OVA_488_) or 647-conjugated OVA protein (OVA_647_) (Invitrogen) was injected into the tail vein, subcutaneous region, or peritoneal cavity of mice. FITC-conjugated dextran (M.W.; 2,000 kDa) (Sigma-Aldrich) was injected into tail vein. Thymic low-density cells were isolated at 4 hrs after OVA protein injection and analyzed by FCM. In another experiments, thymi were subjected to the immunofluorescent staining to observe the localization of antigen.

### *In vitro* antigen uptake assay

Thymic low-density cells were incubated with 10 μg/ml OVA_647_ in RPMI1640 at 37°C for 20 min. Uptake of OVA_647_ was analyzed by FCM after being stained with anti-CD11c and anti-Sirpα Abs.

### Generation of bone marrow chimeric (BMC) mice

Bone marrow cells were collected from WT and DO11.10 mice. DO11.10 bone marrow cells were mixed with WT bone marrow cells at the ratios of 10%, 5%, 2.5%, and 1%. WT mice were irradiated to 7 Gy by using an x-ray irradiator, RX-650 (Faxitron X-ray), and were subsequently given 10^7^ mixed bone marrow cells intravenously.

### Effects of OVA antigen on TCR transgenic thymocytes

OT-I, OT-II, and DO11.10 transgenic mice with or without *CCR2* gene deficiency, and BMC mice were administered with OVA protein (Sigma-Aldrich) or heat-aggregated OVA protein (80°C for 10 min) in PBS through the tail vein. At the indicated time points after the treatment, thymocytes were collected and analyzed by FCM. In some experiments, the thymus was subjected to immunofluorescence analysis.

### *In vitro* differentiation of Tregs

Thymocytes were isolated from mice 2 days after the last OVA protein injection and were incubated with 5 ng/ml rIL-7 in the presence or the absence of the indicated concentrations of rIL-2 in RPMI1640 supplemented with 10% FCS and 50 μM 2-mercaptoethanol at 37°C for 24 hrs. In some experiments, cells were pre-incubated with 2 μg/ml anti-CD25 or anti-CD122 blocking mAbs and were subsequently stimulated with rIL-2 in the presence of the same mAbs at 1 μg/ml.

### Tumor injection

Five ×10^5^ of WT Col26 or their transfectant clones in 200 μl PBS were injected into the dorsal subcutaneous space of WT, DO11.10, or DO11.10/CCR2^−/−^ mice.

### Statistical analysis

Data were analyzed statistically using one-way ANOVA followed by the Turkey-Kramer test. Mann-Whitney's *U* test or Kruskal-Wallis test was used in the instances when the data were not normally distributed. *p*<0.05 was considered statistically significant.

## Results

### Thymic Sirpα^+^ cDCs are a murine counterpart of human DC-SIGN^+^ DCs and can efficiently uptake antigens leaking inside the IVRs

**Figure 1:**
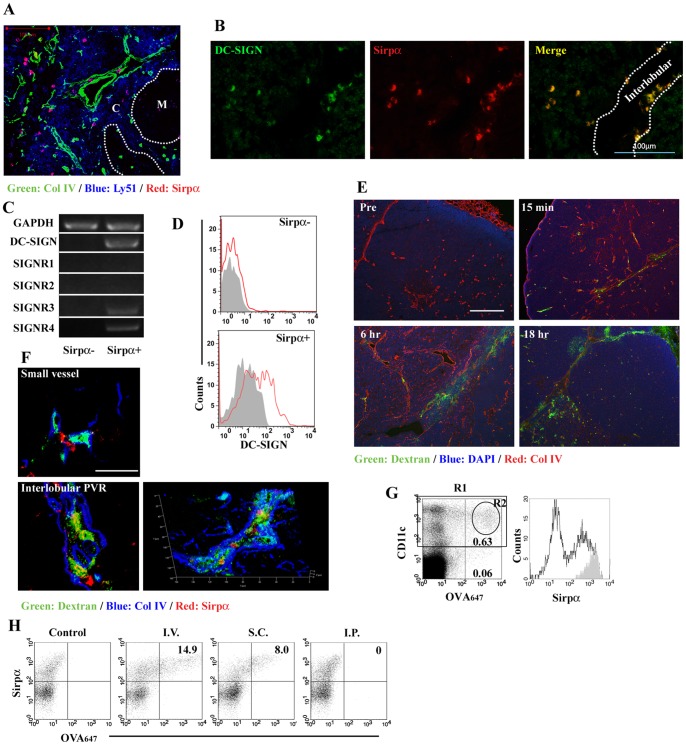
Thymic Sirpα^+^DC-SIGN^+^ cDCs can uptake antigens, which leak inside the IVRs. (**A**) Triple-color fluorescent image with the combination of Col IV (green), Ly-51 (blue), and Sirpα (red) in mouse thymic tissue. Dashed lines indicate the boundary between cortex (C) and medulla (M). Scale bar, 100 µm. (**B**) A double-color fluorescent immunostaining for DC-SIGN (green) and Sirpα (red) in human thymic tissue. Dashed lines indicate the outer margin of thymic lobule. Scale bar, 100 µm. (**C**) Thymic B220^−^CD11c^high^Sirpα^−^ (Sirpα^−^) and B220^−^CD11c^high^Sirpα^+^ (Sirpα^+^) cDCs were sorted (Sirpα^−^ purity, 97.8%; Sirpα^+^ purity, 97.1%) by using FACSAria (BD Biosciences), from thymic low-density cells isolated from 10 mice. Expression of DC-SIGN, SIGNR1, SIGNR2, SIGNR3, and SIGNR4 was determined by RT-PCR. GAPDH served as an internal positive control. RT-PCR analysis was repeated three times. (**D**) DC-SIGN expression on CD11c^high^Sirpα^−^ and CD11c^high^Sirpα^+^ cDCs. Gray-filled and red-open histograms indicate the results obtained using isotype control and specific mAbs for DC-SIGN, respectively. (**E**) Thymi were collected at the indicated time points after FITC-conjugated dextran was intravenously injected into WT mice. Triple-color fluorescent images with the combination of dextran (green), DAPI (blue), and Col IV (red) are shown here. Scale bar, 200 µm. (**F**) Triple-color fluorescent images with the combination of dextran (green), Col IV (blue), and Sirpα (red) at 6 hrs after dextran injection are shown here. The confocal images in the parenchyma nearby small vessel and inside the IVRs, and superposed 3-D image inside the IVRs are shown in the upper and lower left, and lower right panels, respectively. Scale bars, 50 µm. (**G**) Uptake of OVA_647_ in low density cells at 4 hrs after injection. Percentage of CD11c (+) OVA_647_ (+) and CD11c (−) OVA_647_ (+) region are shown. Expression of Sirpα molecule in region 1 (R1) and R2 are represented by unfilled and gray-filled histogram, respectively. (**H**) Uptake of OVA_647_ at 4 hrs after intravenous, subcutaneous, or intraperitoneal injection. Percentage of Sirpα (+) OVA_647_ (+) region in thymic CD11c^high^ DCs is shown in each panel. PBS was injected as a control. Representative results from three independent experiments are shown here.

In WT mouse-derived thymus, thymic Sirpα^+^ cDCs were disseminated in the cortex and inside the IVRs, separated by two Col IV^+^ basement membranes (Fig. 1A). Consistent with the report that DC-SIGN^+^ DCs were present in the cortex and interlobular regions of human thymus [19], [20], we detected DC-SIGN^+^ cells in the parenchyma and interlobular region of human thymus (left panel in Fig. 1B). A double-color immunofluorescence analysis further revealed that most DC-SIGN^+^ cells co-expressed human Sirpα molecule (Fig. 1B). Mouse thymic Sirpα^+^ cDCs, but not Sirpα^−^ cDCs, expressed the transcripts of DC-SIGN, SIGNR3, and SIGNR4 (Fig. 1C) and DC-SIGN molecule on their cell surface (Fig. 1D), indicating that mouse thymic Sirpα^+^ cDCs can be a counterpart of human DC-SIGN^+^ DCs. When intravenously injected, FITC-conjugated dextran polysaccharide leaked through the vessel wall and pervaded inside the IVRs, but its leakage from small vessels was hardly observed in the parenchyma (Fig. 1E). Sirpα^+^ cDCs in the IVRs, but not those in close proximity to the small vessels in the cortex (upper panel in Fig. 1F), efficiently captured dextran as revealed by confocal 2-D and superposed 3-D images (lower panels in Fig. 1F). Intravenously injected OVA protein was also captured mainly by CD11c+ DC population, especially by those expressing Sirpα (Fig. 1G). Moreover, thymic Sirpα^+^ cDCs captured OVA protein, when it was administered subcutaneously, but not intraperitoneally (Fig. 1H). In contrast, CD11b^+^MHC class II^low^F4/80^+^ thymic macrophages failed to take in intravenously injected OVA protein (Fig. S1).

### Antigen uptake by Sirpα^+^ cDCs was impaired in CCR2-deficient thymus

**Figure 2:**
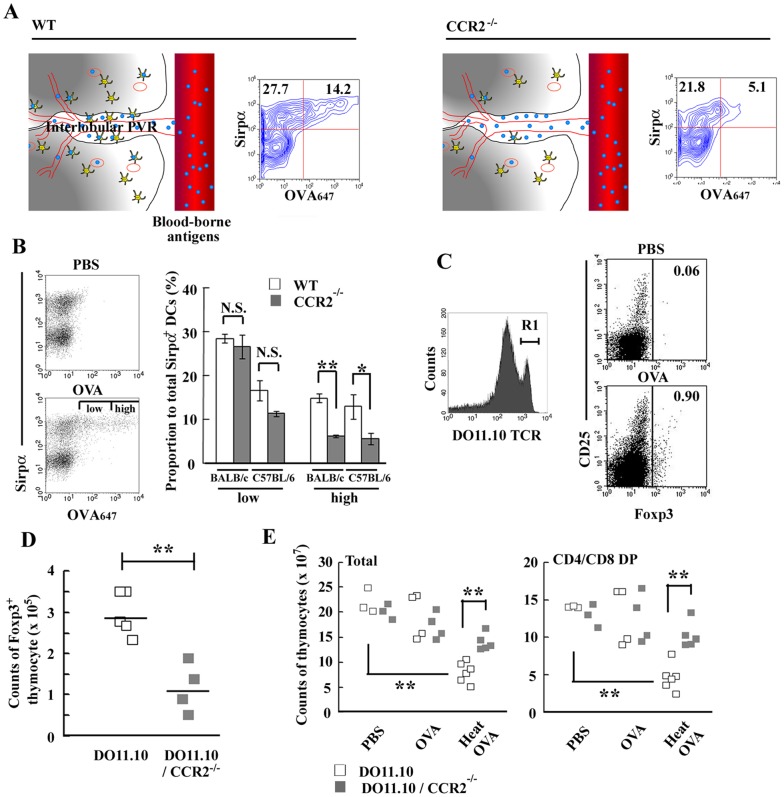
Mice deficient in *CCR2* gene exhibited reduced Treg differentiation and negative selection in the thymus. (**A**) Schematic representation of localization and function of thymic Sirpα^+^ cDCs. OVA protein uptake by CD11c^high^Sirpα^+^ cDCs in WT and CCR2^−/−^ thymus at 4 hrs after injection are shown. Percentage of Sirpα (+) OVA_647_ (−) and Sirpα (+) OVA_647_ (+) region are shown in each panel. (**B**) The uptake of OVA_647_ by CD11c^+^Sirpα^+^ cDCs derived from C57BL/6 thymi at 4 hrs after injection is shown in left panel. Sirpα^+^ cDCs capturing OVA_647_ were separated into two groups according to the efficiency of OVA_647_ uptake; low and high. Percentages of cells in low and high regions in WT and CCR2^−/−^ mice in BALB/c and C57BL/6 strain are shown in the right panel. Data represent mean ± SD from five and three independent experiments in BALB/c and C57BL/6 mice, respectively. (**C**) Expression of CD25 and Foxp3 on DO11.10^high^ (R1) thymocytes at 2 days after OVA protein injection. Percentage of Foxp3 (+) region is shown in each panel. Representative results from three independent experiments are shown. (**D**) The numbers of Foxp3^+^ mature thymocytes were determined on DO11.10 and DO11.10/CCR2^−/−^ mice at 2 days after OVA injection. Each symbol represents an individual mouse. Small horizontal lines indicate the mean. (**E**) Clonal deletion of thymocytes after OVA injection. Two mg OVA protein or heat-aggregated OVA protein were intravenously injected into DO11.10 mice. At 2 days after injection, the numbers of total thymocytes (left graph) and DP thymocytes (right graph) were determined on DO11.10 and DO11.10/CCR2^−/−^ mice. Each symbol represents an individual mouse. **, *p*<0.01. *, *p*<0.05. N.S., no significant difference.

We previously reported that CCR2^−/−^ mouse-derived thymus preserves the architecture of cortical TECs and mTECs with a selective decrease in the number of CD11c^+^CD11b^+^CD8α^−^Sirpα^+^ cDCs [14]. Moreover, *CCR2* gene deficiency caused aberrant localization of Sirpα^+^ cDCs [14], especially in the IVRs where Sirpα^+^ cDCs can efficiently capture antigens, which leaked from bloodstream (Fig. 1 and illustrated in Fig. 2A). Furthermore, *CCR2* gene ablation significantly decreased the proportion of Sirpα^+^ cDCs capturing OVA protein among total intrathymic CD11c^high^ DCs (Fig. 2A). After intravenous OVA protein injection, Sirpα^+^ cDCs of WT mice contained a substantial proportion of the cells with a higher uptake of OVA protein, but this population was markedly decreased by *CCR2* gene ablation in both BALB/c and C57BL/6 mouse strains (Fig. 2B). In contrast, the cells with a lower OVA protein uptake were present with similar proportions in both wild-type and CCR2^−/−^ thymus. Moreover, CCR2^−/−^ Sirpα^+^ cDCs had *in vitro* a similar level of capacity to capture OVA protein as WT cells did (Fig. S2). Thus, intrathymic OVA uptake by Sirpα^+^ cDCs was impaired in CCR2^−/−^ mouse-derived thymus, arising from their reduced number and inappropriate localization in the thymus.

### CCR2-deficient thymus is selectively defective in the tolerogenic roles of Sirpα^+^ cDCs with reduced intrathymic Treg differentiation and negative selection against blood-borne antigens

To examine the function of Sirpα^+^ cDCs in central tolerance against blood-borne antigens, OVA protein was intravenously injected into DO11.10 mice. OVA protein injection induced the generation of Foxp3^+^ Tregs in DO11.10^high^ mature thymocytes (Fig. 2C), which predominantly express a single TCR transgene [21]. In contrast, an increment of Foxp3^+^ thymocytes was markedly attenuated by the absence of *CCR2* gene (Fig. 2D). It was reported that CD25^high^ natural Tregs were spontaneously differentiated from DO11.10^negative to low^ endogenous TCR-rearranged thymocytes [21]. However, OVA protein injection failed to increase Foxp3 expression in this population (Fig. S3). Likewise, OVA protein injection enhanced the proportion of Foxp3^+^ cells in OT-II mice (Fig. S4), but not in OT-I mice of C57BL/6 background (data not shown). Moreover, IL-17 was also detected intracellularly in DO11.10^negative to low^ thymocytes consistent with the previous report [22], but OVA injection failed to increase DO11.10^high^ thymocytes expressing IL-17 (Fig. S5). When we injected the heat-aggregated OVA protein, which forms a highly immunogenic amyloid-like structure [23], [24], it markedly decreased the number of thymocytes in DO11.10 mice, especially those in CD4^+^CD8^+^ (DP) stage, and this reduction was abrogated in DO11.10/CCR2^−/−^ thymus (Fig. 2E). Given selective decreases in numbers of Sirpα^+^ cDCs and their lower capacity of antigen uptake in the thymus of CCR2^−/−^ mice, thymic Sirpα^+^ cDCs can essentially contribute to the induction of Treg differentiation and negative selection, depending on the immunogenicity of the presented antigens.

### Blood-borne antigen can induce intrathymic Treg generation in collaboration with IL-2-mediated signal in a physiological condition

**Figure 3:**
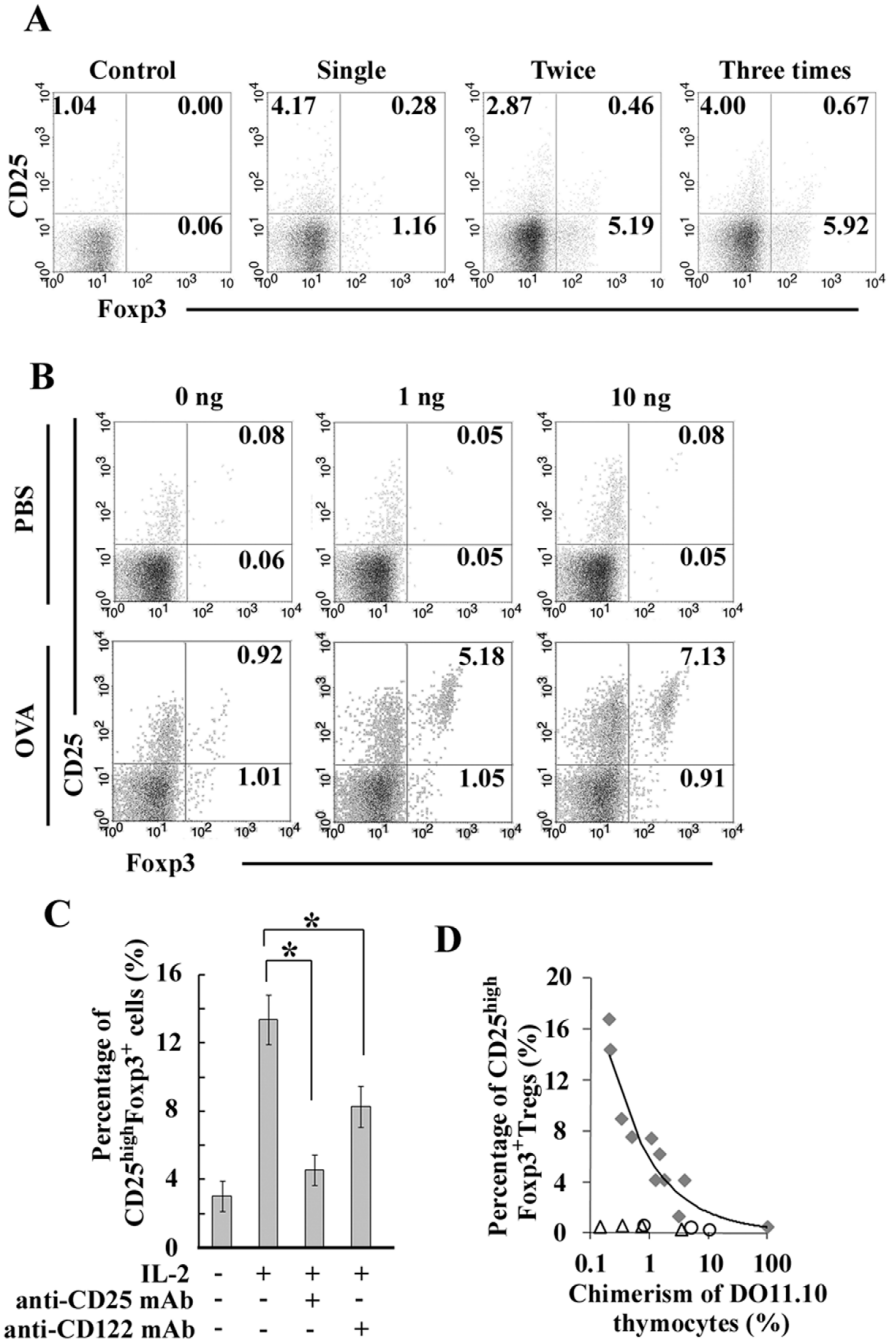
Antigen-specific Treg generation by Sirpα^+^ cDCs in collaboration with IL-2 in a physiological condition. (**A**) At 2 days after the last intravenous injection of OVA protein (2 mg) into DO11.10 mice, Foxp3 and CD25 expression on DO11.10^high^ (R1) thymocytes was examined. PBS was injected as a control. Percentages of CD25^high^Foxp3^−^, CD25^high^Foxp3^+^, and CD25^low^Foxp3^+^ cells are shown in each panel. Representative results from three independent experiments are shown**.** (**B**) Expression of CD25 and Foxp3 on DO11.10^high^ mature thymocytes after stimulation with IL-2 *in vitro*. Percentages of CD25^high^Foxp3^+^ and CD25^low^Foxp3^+^ cells are shown in each panel. Representative results from three independent experiments are shown. (**C**) At 2 days after twice intravenous injection of OVA protein, thymocytes were cultured with 2 ng/ml IL-2 for 24 hrs in the presence or absence of each blocking Ab. Percentage of CD25^high^Foxp3^+^ cells was determined. Data represent mean ± SD from three independent experiments. *, *p*<0.01. (**D**) Percentage of CD25^high^Foxp3^+^ cells among DO11.10^high^ thymocytes in BMC mice was analyzed at 2 days after intravenous injection with OVA protein. Gray-filled symbol represents OVA protein-injected mice. Data from non-BMC DO11.10 mouse are shown as a symbol of 100% chimerism of DO11.10 thymocytes. PBS (unfilled circle) and BSA (unfilled triangle) were injected as controls. Percentage of chimerism  = % of DO11.10^high^ thymocytes in BMC thymus/% of DO11.10^high^ thymocytes in non-BMC DO11.10 thymus x 100. Each symbol indicates one animal.

The repeated intravenous injection of OVA protein progressively enhanced Foxp3 expression in CD25^−^DO11.10^high^ thymocytes (Fig. 3A). OVA protein injection alone induced immature Foxp3^+^ thymocytes expressing a lower level of CD25 (Fig. 3A), but mature DO11.10^high^CD25^high^Foxp3^+^ Tregs were consistently generated by the subsequent *in vitro* stimulation with IL-2 after the injection with OVA protein but not PBS (Fig. 3B). The *in vitro* stimulation with IL-2 further efficiently increased mature Tregs following the twice injection of OVA protein, and the increase was inhibited by anti-CD25 mAb and to a lesser extent, anti-CD122 blocking mAb (Fig. 3C). Moreover, intravenous injection of OVA protein markedly increased percentage of CD25^high^Foxp3^+^ cells among DO11.10^high^ cell population in BMC mice containing various percentages of DO11.10 thymocytes to polyclonal thymocytes, and the increase was correlated inversely with the chimerism ratios (Fig. 3D). Thus, monoclonal TCR transgenic thymocytes may compete with each other to receive some prerequisite signals such as IL-2 from intrathymic environment during Treg differentiation, as previously reported [25]. Thus, intrathymic presentation of blood-borne antigens which can be selectively captured by thymic Sirpα^+^ cDCs initiates Treg differentiation in collaboration with some factors such as IL-2.

### CCL2-CCR2 axis can enhance the capacity of antigen uptake by Sirpα^+^ cDCs in tumor-bearing mice

**Figure 4:**
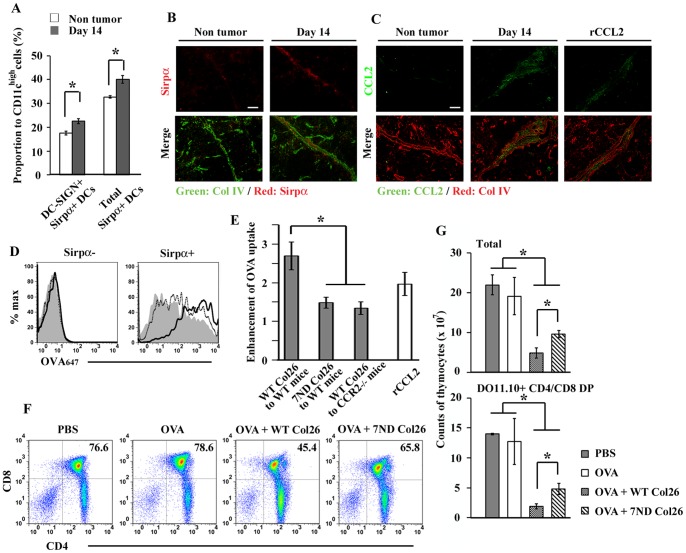
Induction of negative selection by Sirpα^+^ cDCs in tumor-bearing mice. (**A**) Thymus was collected at 14 days after Col26 tumor inoculation. Percentage of DC-SIGN^+^Sirpα^+^ and total Sirpα^+^ cells in thymic CD11c^high^ DCs in WT mice bearing tumor or mice without tumor are shown. (**B**) A double-color fluorescent immunostaining for Sirpα (red) and Col IV (green). Single color image for Sirpα and merged image are shown on the upper and lower, respectively. Scale bar, 50 µm. (**C**) A double-color fluorescent immunostaining for Col IV (red) and CCL2 (green). The right panel shows the thymus which was collected at 1 hr after intravenous injection of rCCL2 (5 μg). Single color image for CCL2 and merged image are shown on the upper and lower, respectively. Scale bar, 50 µm. (**D**) Parental Col26 and Col26-7ND were injected into WT mice. Subsequently, OVA_647_ was intravenously injected at 14 days after tumor inoculation. Uptake of OVA_647_ in Sirpα^−^ (left) and Sirpα^+^ cDCs (right) were analyzed at 4 hrs after OVA injection. Parental Col26-, Col26-7ND-bearing mouse, and mouse without tumor were represented by a solid-lined, dash-lined, and gray-filled histogram, respectively. Representative results from 3 independent experiments are shown here. (**E**) Enhancement of the capability of OVA uptake by Sirpα^+^ cDCs in tumor-bearing mice. Enhancement of OVA uptake  =  MFI of OVA_647_ captured by Sirpα^+^ DCs in WT or CCR2^−/−^ mice bearing parental Col26 or Col26-7ND tumor/that in WT or CCR2^−/−^ mice without tumor. The right unfilled column shows the fold-enhancement of OVA_647_, when OVA_647_ was injected at 1 day after three times of daily injections of rCCL2 (2.5 μg/injection). (**F**) Two mg OVA protein was intravenously injected into DO11.10 mice at 14 days after tumor inoculation and 2 days later, expression of CD4 and CD8 in DO11.10^+^ thymocytes was analyzed. Percentage of DP thymocytes is shown in each panel. Data represent mean ± SD from three independent experiments. (**G**) The numbers of total thymocytes (upper graph) and DP thymocytes (lower graph) are shown. Representative results from three (**B** and **C**) or four (**D** and **F**) independent experiments are shown here. Data represent mean ± SD from four independent experiments (**A**, **E**, and **G**). *, *p*<0.01.

CCR2 expression by Sirpα^+^ cDCs prompted us to examine the function of Sirpα^+^ cDCs in tumor-bearing mice, because CCR2 ligands, particularly CCL2, are abundantly expressed in tumor-bearing mice [26]. When murine colorectal cancer cell line, Col26 was subcutaneously injected into WT mice, the proportions of both DC-SIGN^+^Sirpα^+^ and total Sirpα^+^ cDCs were significantly increased among thymic CD11c^high^ cDC population at 14 days after tumor inoculation (Fig. 4A) and were accumulated in the IVRs in tumor-bearing mice (Fig. 4B) with a marked increase in serum CCL2 (Fig. S6B). Immunofluorescence analysis revealed that CCL2 was intensively detected inside the IVRs in the thymus derived from tumor-bearing mice (Fig. 4C). The carboxy-terminal region of chemokines show a high binding affinity for proteoglycans on the vascular endothelium and in the extracellular matrix [27]. Given the abundance of proteoglycans in the IVRs in the thymus [28], serum CCL2 might be deposited inside the IVRs through binding with proteoglycan after tumor development. This assumption is supported by the observation that CCL2 was detected in the IVRs in non-tumor-bearing mice after intravenous injection of recombinant (r) CCL2 (Fig. 4C). Furthermore, tumor-bearing mice exhibited enhanced intrathymic antigen uptake by Sirpα^+^ cDCs, but not by Sirpα^−^ cDCs, when OVA protein was intravenously injected (Fig. 4D). This enhanced uptake of the intravenously administered antigen was partially retarded when OVA protein was intravenously injected into Col26-7ND tumor-bearing WT mice (Fig. 4E). Col26-7ND produced significantly less mouse CCL2 than the parental cells *in vitro* (Fig. S6A). Moreover, the *in vitro* co-culture of Col26-7ND reduced mouse CCL2 secretion by the parental cells (Fig. S6A). Thus, 7ND protein can inhibit endogenous CCL2 production in a paracrine and/or autocrine manner. Moreover, WT mice receiving the tumor expressing 7ND exhibited reduced serum mouse CCL2 concentration compared with WT mice receiving parental Col26 cells (Fig. S6B). Thus, human 7ND protein could reduce the release of mouse CCL2 by Col26 cells probably in an autocrine manner. Compared with wild-type mice, CCR2^−/−^ mice exhibited depressed uptake of intravenously administered OVA protein even when they were administered with the parental tumor (Fig. 4E). These observations would implicate the involvement of the CCL2-CCR2 axis in the uptake of an intravenously administered antigen by Sirpα^+^ cDCs. Intravenous injection of rCCL2 enhanced *in vivo* the uptake of intravenously injected OVA protein (Fig. 4E). On the contrary, rCCL2 failed *in vitro* to enhance antigen uptake by Sirpα^+^ cDCs (Fig. S2). Thus, CCL2 deposited inside the IVRs can induce the trafficking of Sirpα^+^ cDCs with a capacity to uptake an intravenously administered antigen into the IVRs, resulting in central tolerance.

### Blood-borne antigen can induce intrathymic negative selection in tumor-bearing mice

To examine the tolerogenic event induced by blood-borne antigen, tumor-bearing mice were intravenously injected with OVA protein. After injection, clonal deletion in OVA-specific thymocytes, especially at DP stage, obviously occurred in DO11.10 mice bearing tumor but not in mice without tumor (Fig. 4F and 4G). This negative selection was partially retarded in mice bearing Col26-7ND tumor (Fig. 4G). In contrast, Treg generation was depressed in the mice bearing a parental Col26 tumor (Fig. S7). Thus, tumor development can induce the shift from Treg differentiation to negative selection, together with exaggerated antigen uptake by Sirpα^+^ cDCs.

### Tumor-derived secretory antigen can efficiently induce the intrathymic negative selection

**Figure 5:**
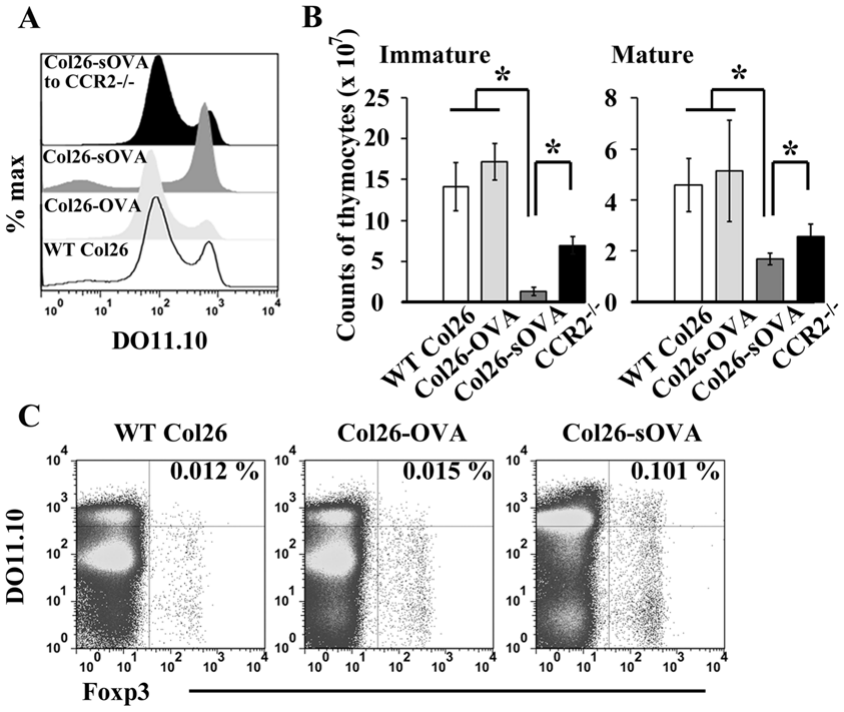
Tumor-derived secretory antigen can efficiently induce the intrathymic negative selection. (**A**) At 14 days after inoculation of parental Col26 and Col26-OVA, or at 22 days after inoculation of Col26-sOVA, each developmental stage of thymocytes was determined on DO11.10 or DO11.10/CCR2^−/−^ mice. Expression of DO11.10 TCR on total thymocytes is shown. (**B**) The numbers of DO11.10^medium^ immature thymocytes (left graph) and DO11.10^high^ mature thymocytes (right graph) were determined on DO11.10 or DO11.10/CCR2^−/−^ mice bearing each tumor. Data represent mean ± SD from three independent experiments. *, *p*<0.01. (**C**) Intrathymic Treg differentiation in the tumor-bearing mice. Percentage of DO11.10^high^Foxp3^+^ Tregs is shown in each panel. Representative results from three (**A** and **C**) independent experiments are shown here.

Finally, we examined whether a tumor-derived antigen can induce the intrathymic Treg differentiation or negative selection. For this purpose, we established two distinct OVA-GFP fusion protein-expressing Col26 clones; Col26-OVA and Col26-sOVA clones, which express the fusion protein only in the cytoplasm and secrete the fusion protein outside of the cells, respectively. Among the obtained Col26-sOVA clones, Col26-sOVA clone 4 secreted OVA-GFP fusion protein into the culture supernatant most efficiently (Fig. S8A) and was therefore used in the following experiments. In DO11.10 mice, Col26 cells and Col26-OVA cells grew at similar rates whereas Col26-sOVA cells grew at slower rates (Fig. S8B) as previously reported [1], [2]. Accordingly, we obtained thymi at 14 days after Col26 or Col26-OVA injection and at 22 days after Col26-sOVA injection, because the tumor sizes were similar at these time points among these three groups. Compared with DO11.10 mice bearing either Col26 or Col26-OVA, both DO11.10^high^ mature thymocytes and DO11.10^medium^ immature thymocytes were decreased in the mice bearing Col26-sOVA (Fig. 5A and 5B). This negative selection was partially retarded in DO11.10/CCR2^−/−^ mice at 22 days after Col26-sOVA injection (Fig. 5A and 5B). In contrast, Treg differentiation in DO11.10^high^ thymocytes was marginal in mice bearing Col26-sOVA (Fig. 5C). Thus, a tumor-specific antigen can induce the intrathymic negative selection but not Treg differentiation, once it is secreted into bloodstream.

## Discussion

Recently, Atibalentja et al reported the involvement of both Sirpα^+^ and Sirpα^−^ cDC in central tolerance to an intravenously administered hen egg white lysozyme (HEL) [29], [30]. Because molecules with a smaller M.W. than BSA (M.W. 67 kDa) can diffuse through the blood-thymus barrier and enter the medulla, HEL (M.W. 14.4 kDa) might be captured by Sirpα^−^ cDC in medulla in the previous studies [29], [30]. In contrast, we observed that the Sirpα^+^ cDCs selectively captured intravenously injected fluorescent dye-conjugated OVA protein (M.W. 45 kDa) and presented it to immature thymocytes in the thymic cortical region. Moreover, thymic Sirpα^+^ cDCs selectively captured mouse IgG (150 kDa) [14], heat-aggregated OVA (too large to measure M.W.) [31], and dextran (M.W. 2,000 kDa), which were injected intravenously. The adult blood–thymus barrier constitutively prevents large-sized antigens from permeating into the thymic parenchyma [32]. However, we observed that dextran with a large molecular weight permeated inside the IVRs, and was subsequently captured efficiently by Sirpα^+^ cDCs therein. Moreover, some Sirpα^+^ cDCs capturing dextran appeared in the thymic cortical parenchyma at 18 hrs after injection (data not shown). These observations may mirror our previous observation that Sirpα^+^ cDCs captured and conveyed OVA protein into the cortical parenchyma [14]. Thus, due to its peculiar localization in the IVR, only Sirpα^+^ cDCs can directly capture large-sized blood-borne antigens, which are unable to permeate into thymic parenchyma.

Thymic Sirpα^+^ cDCs captured OVA protein, when it was administered intravenously or subcutaneously, but not intraperitoneally. Boelaert J et al. previously reported that serum concentration of erythropoietin (M.W. 34 kDa) rose more slowly after subcutaneous injection than after intravenous injection [33]. They further demonstrated that intraperitoneal injection was much less effective in the raising of serum concentration than subcutaneous injection. Given the capacity of Sirpα^+^ cDCs to capture selectively blood-borne antigens, intraperitonal injection of OVA protein may not be able to increase its serum concentration sufficient for Sirpα^+^ cDCs to capture it in the thymus.

We demonstrated that thymic Sirpα^+^ cDCs can capture blood-borne antigens and can initiate the antigen-specific Treg differentiation in a physiological condition. The “two-step model” hypothesis proposed that additional IL-2 signal is a prerequisite for intrathymic Treg differentiation after antigen presentation [34], [35]. Consistent with this assumption, we observed that IL-2 induced Treg precursors to differentiate into mature CD25^high^Foxp3^positive^ Tregs without additional TCR engagement. Moreover, the differentiation of CD25^+^Foxp3^+^ mature Tregs was more efficient in an intraclonal low-competitive condition as revealed by the experiment using mixed BMC. Thus, Sirpα^+^ cDCs can capture a blood-borne antigen and present it to the thymocytes, leading to the efficient generation of Tregs in collaboration with several factors including IL-2 in a physiological condition.

Accumulation of Sirpα^+^ cDCs in IVRs can result in more efficient capture of an intravenously injected antigen and subsequent intrathymic negative selection. Subcutaneous tumor formation of Col26 increased the serum concentration of CCL2, which was deposited in IVRs and attracted Sirpα^+^ cDCs therein. Consequently, Sirpα^+^ cDCs can capture efficiently a secretory OVA protein and can induce intrathymic negative selection. However, we cannot exclude the possibility that the secretion of a tumor-specific antigen may induce the expression of additional factors, which may induce the intrathymic negative selection in cooperation with CCR2-expressing Sirpα^+^ cDCs.

Consistent with the previous reports [1], [2], OVA-secreting tumors grew more slowly in DO11.10 mice than OVA-non-secreting ones did, in the early phase, despite the absence of *in vitro* growth rate difference between OVA-secreting and OVA-non-secreting cells (data not shown). Thus, when a tumor is impalpable, a secretory OVA protein may induce intratumoral expansion of DO10.11 T lymphocytes, which can exhibit immune surveillance against an OVA-expressing tumor. In contrast, when a tumor becomes palpable, OVA protein may circulate systemically to reach thymus. Thymic Sirpα^+^ cDCs can uptake OVA protein and subsequently induce intrathymic negative selection in DO11.10 mice. Thus, if thymic Sirpα^+^ cDCs can be well tuned, the secretory tumor-specific antigens may become more beneficial for the immune surveillance against a tumor.

Herein, we have unraveled a pivotal role for thymic Sirpα^+^ cDC subset in the distinct surveillance against blood-borne antigens in the central tolerance system. Moreover, we proved that an inflammatory chemokine receptor, CCR2, have profound effects on the tolerogenic capacity of thymic Sirpα^+^ cDCs. We recently demonstrated that ablation of the *CCR2* gene exacerbated the pathology of polyarthritis spontaneously developed in IL-1 receptor antagonist-deficient mice [36], suggesting its immune regulatory roles in autoimmunity. Moreover, mouse thymic Sirpα^+^ cDCs can be a counterpart of human thymic DC-SIGN^+^Sirpα^+^ DCs, the cells that are presumed to be involved in thymocyte selection in humans [19], [20]. Thus, in both mice and humans, this cell population may regulate immune responses against blood-borne antigens and eventually have pathophysiological roles in the various disorders representing abnormal immune status, including cancer, autoimmune diseases, and allergy.

## Supporting Information

Figure S1 **Uptake of blood-borne antigen by thymic CD11b^+^ DCs, but not macrophages.** CD11b^+^MHC class II^+^ DCs (R1) and F4/80^+^ macrophages (R3) among CD11b^+^MHC class II^−^ cells (R2) were gated to analyze the uptake of OVA_488_ (red line-histogram). Autofluorescence of each population is represented by gray-filled histogram. Representative results from two independent experiments are shown here.
(TIF)

Figure S2 ***In vitro*** **antigen uptake by thymic Sirpα^+^ cDCs.** *In vitro* uptake of OVA_647_ by thymic Sirpα^+^ cDCs which were isolated from WT, CCR2^−/−^, or WT mice after three times of daily injections of rCCL2 (2.5 μg/injection) was examined. MFI of OVA_647_ captured by CD11c^high^Sirpα^+^ cDCs was determined and mean ± SD from three independent experiments was shown.
(TIF)

Figure S3 **Natural DO11.10^negative to low^ Tregs.** At 2 days after DO11.10 mice received intravenous injection of OVA protein (2 mg), CD25 and Foxp3 expression on DO11.10^negative to low^ (R1) thymocytes was examined. PBS was injected as a control. The proportion of CD25^high^Foxp3^+^ and CD25^low^Foxp3^+^ cells is shown in each panel. Representative results from three independent experiments are shown here.
(TIF)

Figure S4 **Induction of Treg differentiation in C57BL/6 background.** At 2 days after intravenous injection of OVA protein (2 mg) into OT-II mice, expression of CD25 and Foxp3 on Vα2 TCR^high^ thymocytes was analyzed. PBS was injected as a control. Percentage of Foxp3 (+) region is shown in each panel. Representative results from three independent experiments are shown here.
(TIF)

Figure S5 **Intrathymic Th17 differentiation.** At 2 days after twice intravenous injection of OVA protein (2 mg), total thymocytes were stimulated with PMA (50 ng/ml) and ionomycin (1 μg/ml) in the presence of GolgiStop Protein Transport Inhibitor (BD Biosciences) for 6 hrs. Expression of IL-17 and Foxp3 were analyzed by FCM, using Mouse Th17/Treg Phenotyping Kit (BD Biosciences). Percentage of IL-17 (+) Foxp3 (−), IL-17 (+) Foxp3 (+), and IL-17 (−) Foxp3 (+) region are shown in each panel. Representative results from three independent experiments are shown here.
(TIF)

Figure S6 **Human 7ND protein reduces the release of mouse CCL2 by Col26 cells in an autocrine manner.** (A) Either parental Col26 or Col26-7ND (1×10^5^) or the mixture of these cells (2×10^5^) at a ratio of 1 to 1 was suspended in 1 ml of culture medium. Culture supernatants were collected 2 days after the incubation and concentration of mouse CCL2 in the supernatant was determined by using mouse CCL2/JE/MCP-1 immunoassay kit (R&D Systems), which do not show cross-reactivity against human CCL2. (B) Parental Col26 or Col26-7ND cells were injected into WT mice. Subsequently, serum concentration of mouse CCL2 was determined at 14 days after tumor inoculation. Serum CCL2 was not detected (N.D.) in mice without tumor. Data represent mean ± SD from three independent experiments. *, *p*<0.05. **, *p*<0.01.
(TIF)

Figure S7 **Defect in intrathymic Treg generation in mice bearing tumor.** Two mg OVA protein was intravenously injected into DO11.10 mice at 14 days after tumor inoculation. At 2 days after the injection, the number of DO11.10^high^Foxp3^+^ Tregs was determined. Data represent mean ± SD from three independent experiments. *, *p*<0.01.
(TIF)

Figure S8 **Growth of Col26 secreting OVA protein was retarded in DO11.10 mice.** (**A**) Col26-OVA and 4 clones of Col26-sOVA were established. Culture supernatant was collected after the incubation of 1×10^5^ cells in 1 ml for 2 days. Concentration of OVA protein in the supernatant was determined. (**B**) Tumor size was measured at every two days after subcutaneous injection of 5×10^5^ cells into DO11.10 mice. The growth of parental Col26, Col26-OVA, and Col26-sOVA cells were represented by unfilled, gray-filled, and black-filled symbols, respectively. Tumor volume (mm^3^)  =  Length x Width x Depth/2. Data represent mean ± SD from three independent experiments.
(TIF)
