# Supplementary figures and images for: Novel Process of Intrathymic Tumor-Immune Tolerance through CCR2-Mediated Recruitment of Sirpα+ Dendritic Cells: A Murine Model
Source: PLoS One. 2012 Jul 16;7(7):e41154. doi: 10.1371/journal.pone.0041154 (PMC3397991; doi:10.1371/journal.pone.0041154)

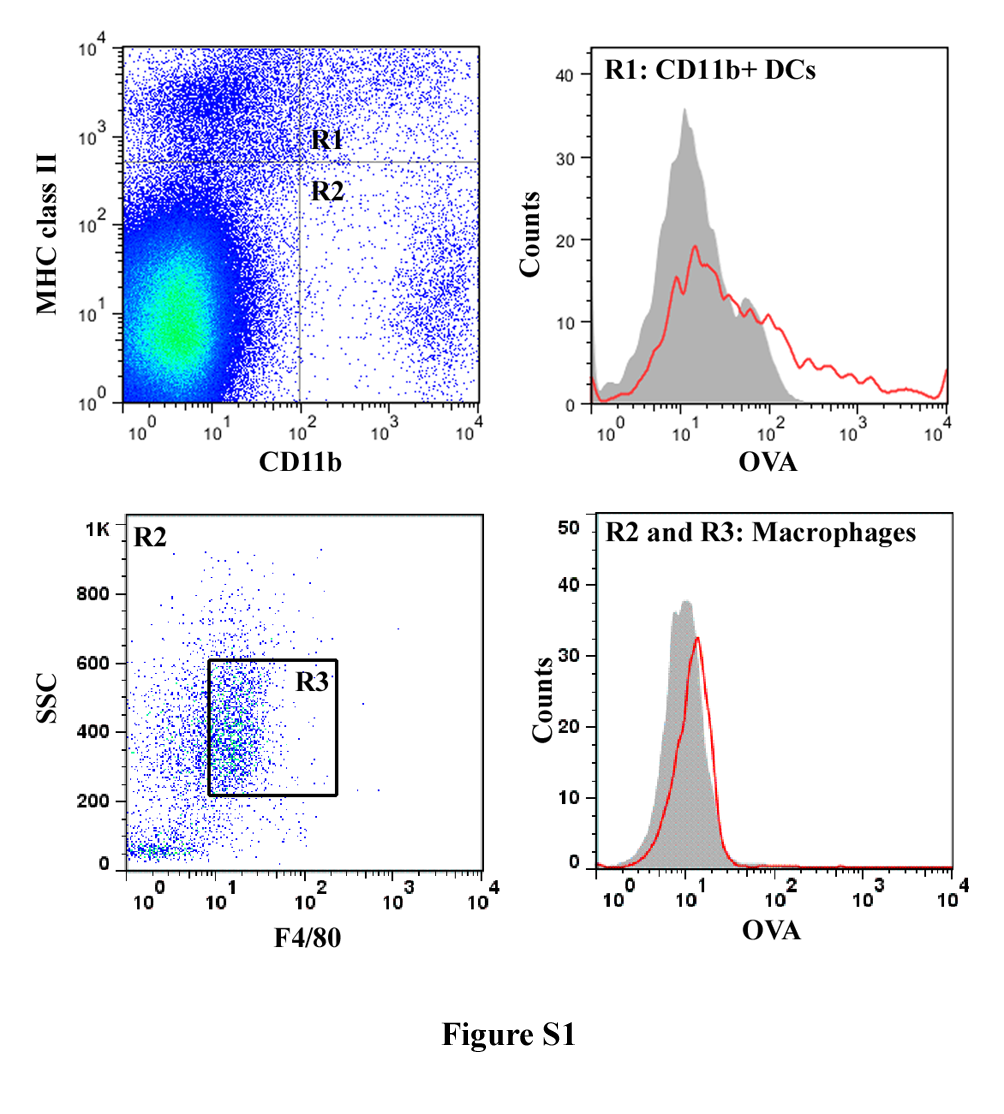

Supplement: Figure S1 — Uptake of blood-borne antigen by thymic CD11b+ DCs, but not macrophages. CD11b+MHC class II+ DCs (R1) and F4/80+ macrophages (R3) among CD11b+MHC class II− cells (R2) were gated to analyze the uptake of OVA488 (red line-histogram). Autofluorescence of each population is represented by gray-filled histogram. Representative results from two independent experiments are shown here. (TIF) [file pone.0041154.s001.tif]

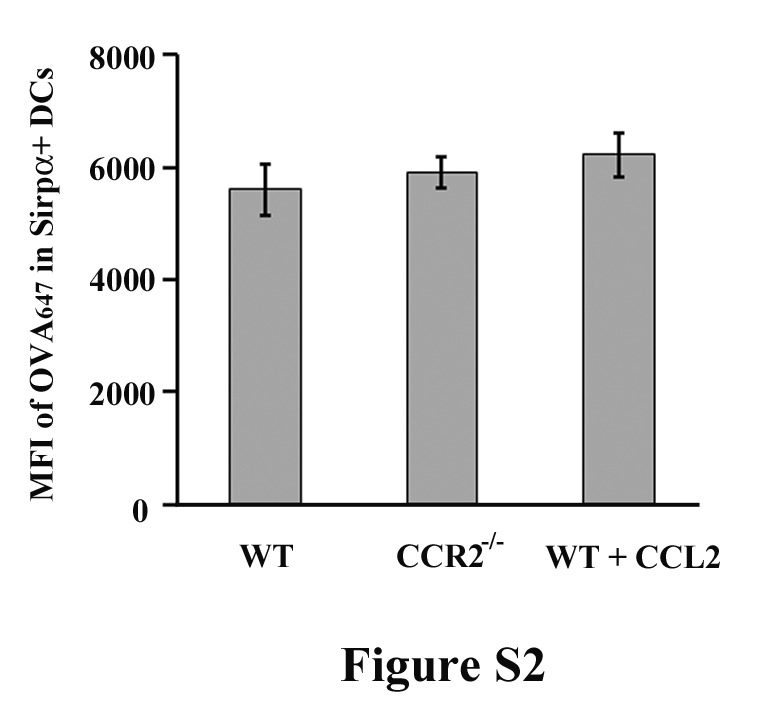

Supplement: Figure S2 — In vitro antigen uptake by thymic Sirpα+ cDCs. In vitro uptake of OVA647 by thymic Sirpα+ cDCs which were isolated from WT, CCR2−/−, or WT mice after three times of daily injections of rCCL2 (2.5 μg/injection) was examined. MFI of OVA647 captured by CD11chighSirpα+ cDCs was determined and mean ± SD from three independent experiments was shown. (TIF) [file pone.0041154.s002.tif]

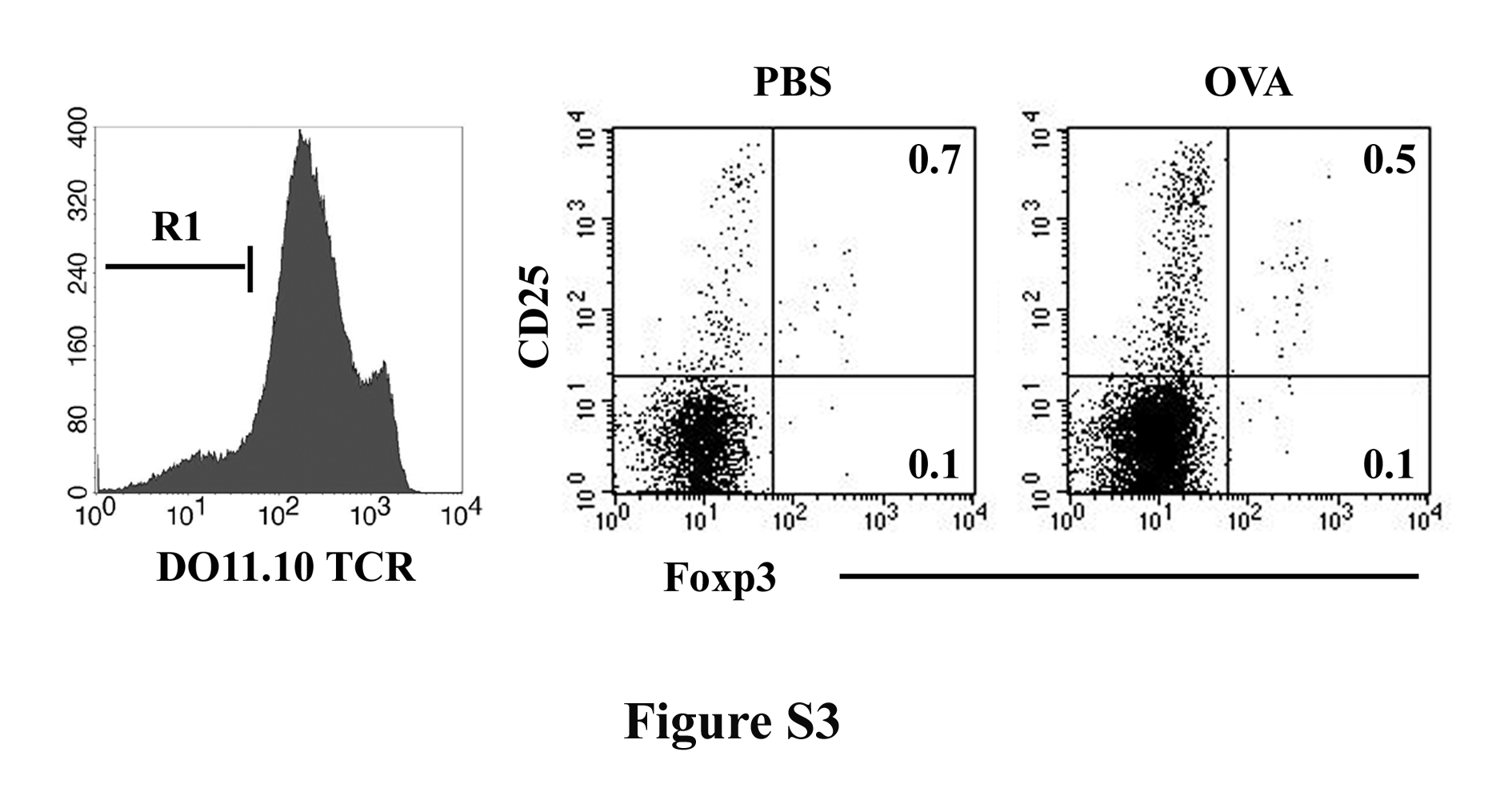

Supplement: Figure S3 — Natural DO11.10negative to low Tregs. At 2 days after DO11.10 mice received intravenous injection of OVA protein (2 mg), CD25 and Foxp3 expression on DO11.10negative to low (R1) thymocytes was examined. PBS was injected as a control. The proportion of CD25highFoxp3+ and CD25lowFoxp3+ cells is shown in each panel. Representative results from three independent experiments are shown here. (TIF) [file pone.0041154.s003.tif]

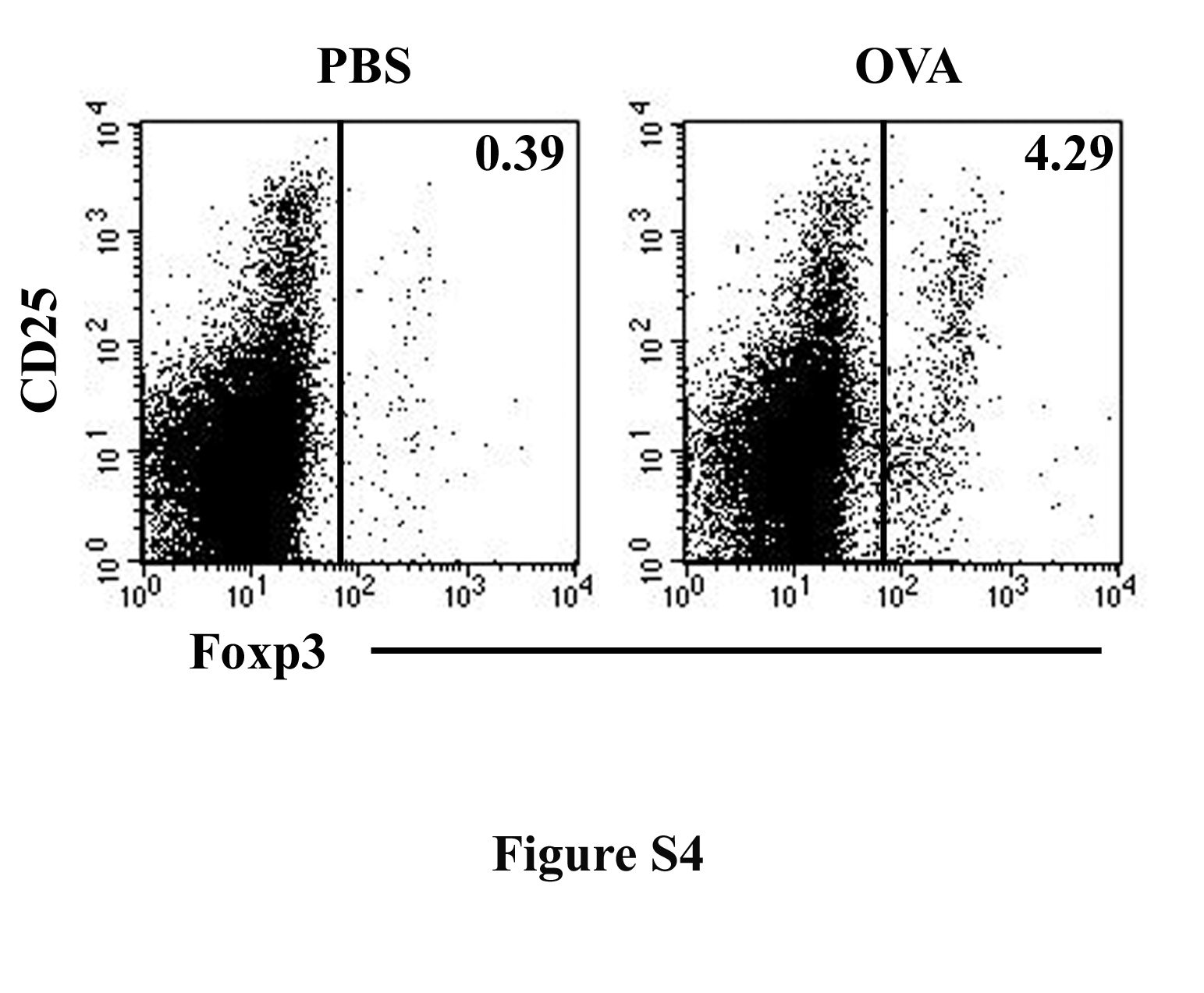

Supplement: Figure S4 — Induction of Treg differentiation in C57BL/6 background. At 2 days after intravenous injection of OVA protein (2 mg) into OT-II mice, expression of CD25 and Foxp3 on Vα2 TCRhigh thymocytes was analyzed. PBS was injected as a control. Percentage of Foxp3 (+) region is shown in each panel. Representative results from three independent experiments are shown here. (TIF) [file pone.0041154.s004.tif]

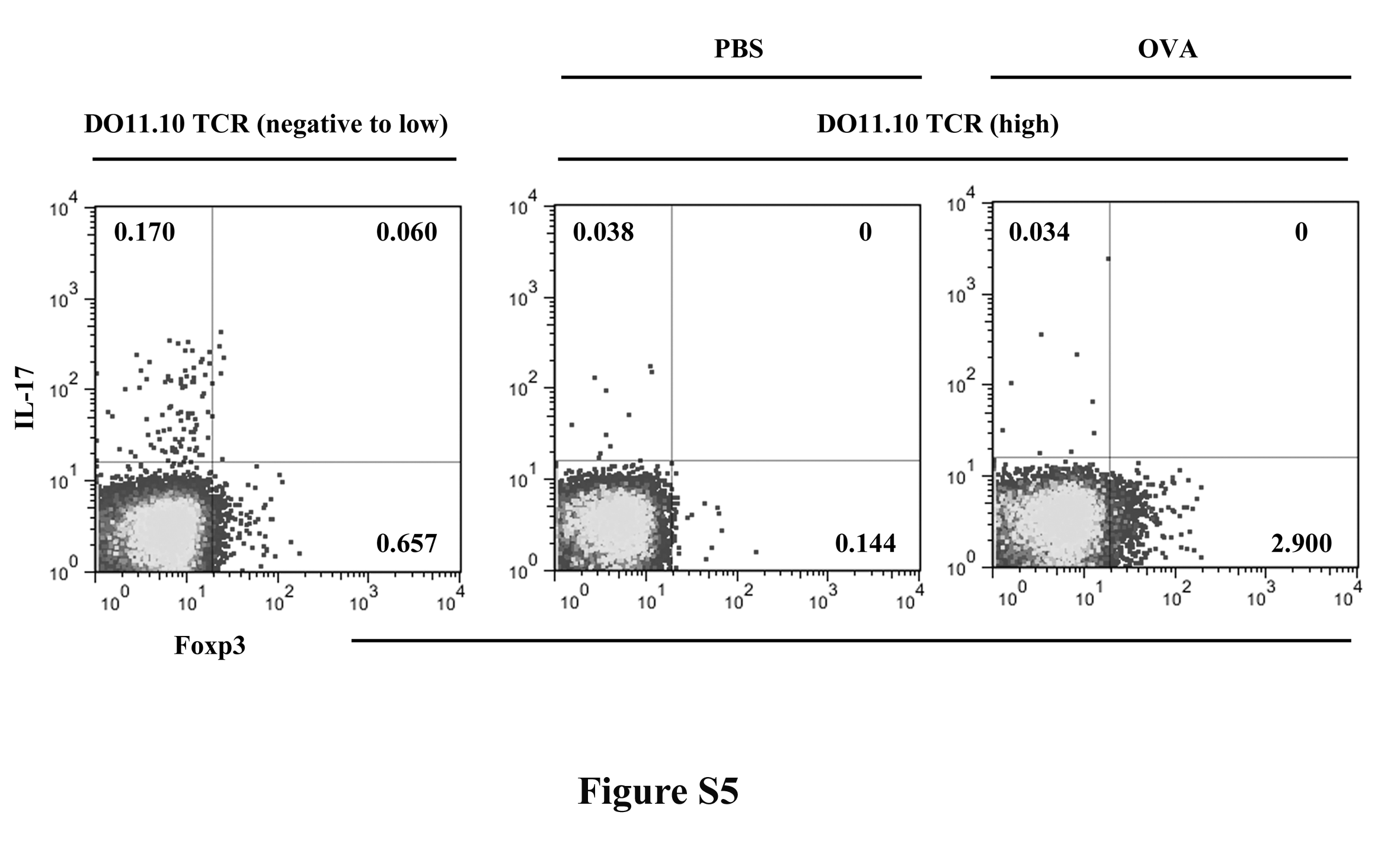

Supplement: Figure S5 — Intrathymic Th17 differentiation. At 2 days after twice intravenous injection of OVA protein (2 mg), total thymocytes were stimulated with PMA (50 ng/ml) and ionomycin (1 μg/ml) in the presence of GolgiStop Protein Transport Inhibitor (BD Biosciences) for 6 hrs. Expression of IL-17 and Foxp3 were analyzed by FCM, using Mouse Th17/Treg Phenotyping Kit (BD Biosciences). Percentage of IL-17 (+) Foxp3 (−), IL-17 (+) Foxp3 (+), and IL-17 (−) Foxp3 (+) region are shown in each panel. Representative results from three independent experiments are shown here. (TIF) [file pone.0041154.s005.tif]

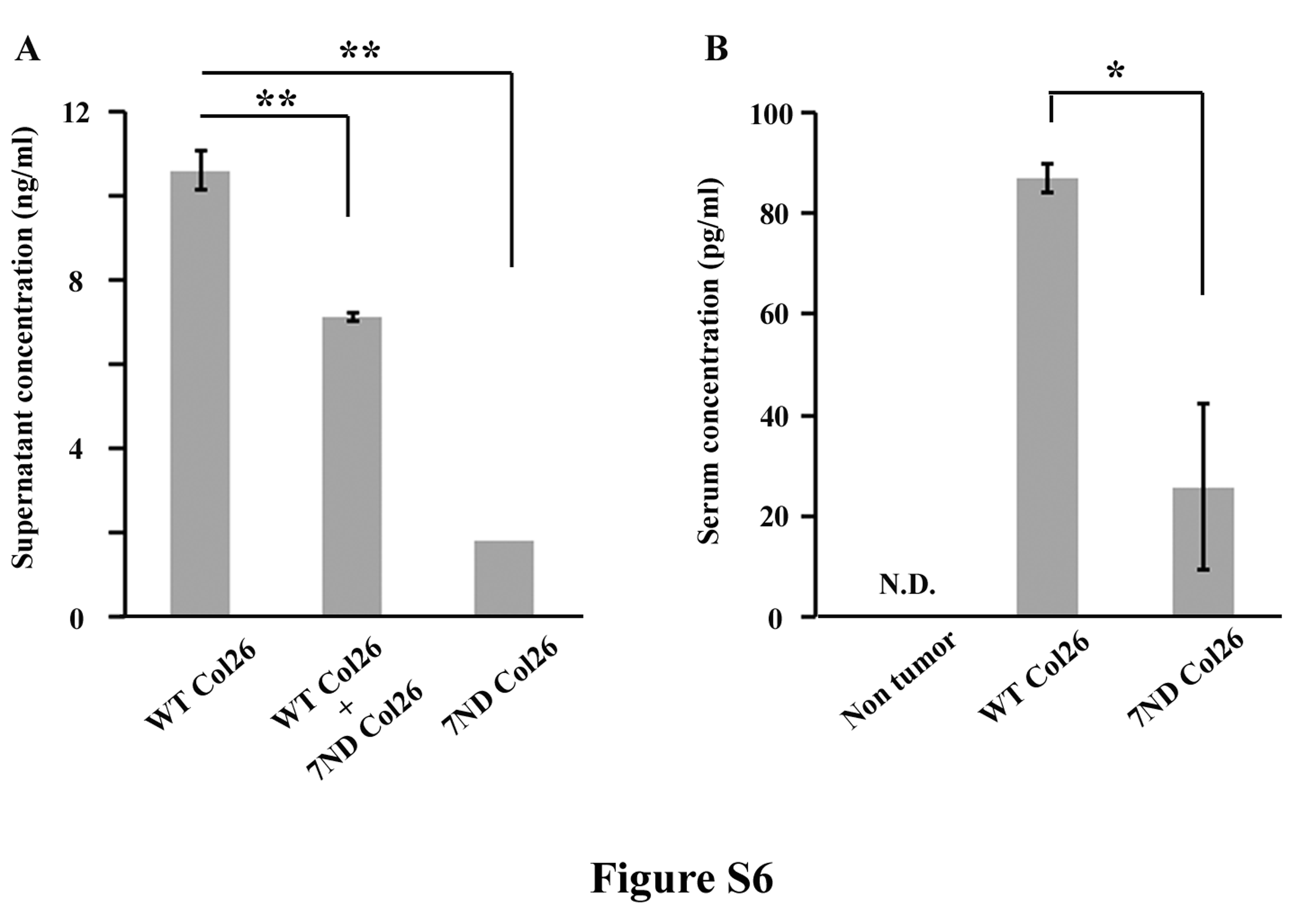

Supplement: Figure S6 — Human 7ND protein reduces the release of mouse CCL2 by Col26 cells in an autocrine manner. (A) Either parental Col26 or Col26-7ND (1×10^5^) or the mixture of these cells (2×10^5^) at a ratio of 1 to 1 was suspended in 1 ml of culture medium. Culture supernatants were collected 2 days after the incubation and concentration of mouse CCL2 in the supernatant was determined by using mouse CCL2/JE/MCP-1 immunoassay kit (R&D Systems), which do not show cross-reactivity against human CCL2. (B) Parental Col26 or Col26-7ND cells were injected into WT mice. Subsequently, serum concentration of mouse CCL2 was determined at 14 days after tumor inoculation. Serum CCL2 was not detected (N.D.) in mice without tumor. Data represent mean ± SD from three independent experiments. *, p<0.05. **, p<0.01. (TIF) [file pone.0041154.s006.tif]

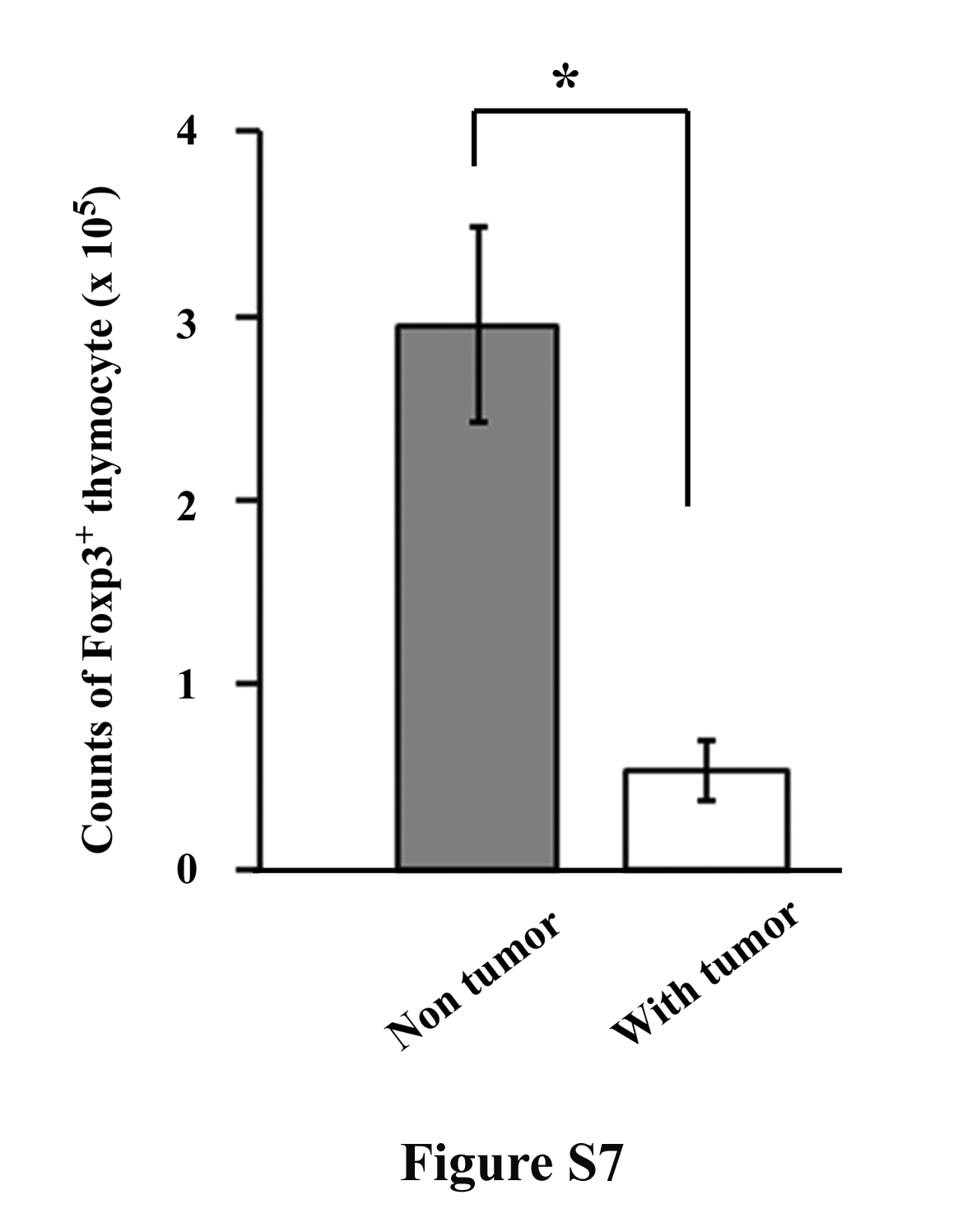

Supplement: Figure S7 — Defect in intrathymic Treg generation in mice bearing tumor. Two mg OVA protein was intravenously injected into DO11.10 mice at 14 days after tumor inoculation. At 2 days after the injection, the number of DO11.10highFoxp3+ Tregs was determined. Data represent mean ± SD from three independent experiments. *, p<0.01. (TIF) [file pone.0041154.s007.tif]

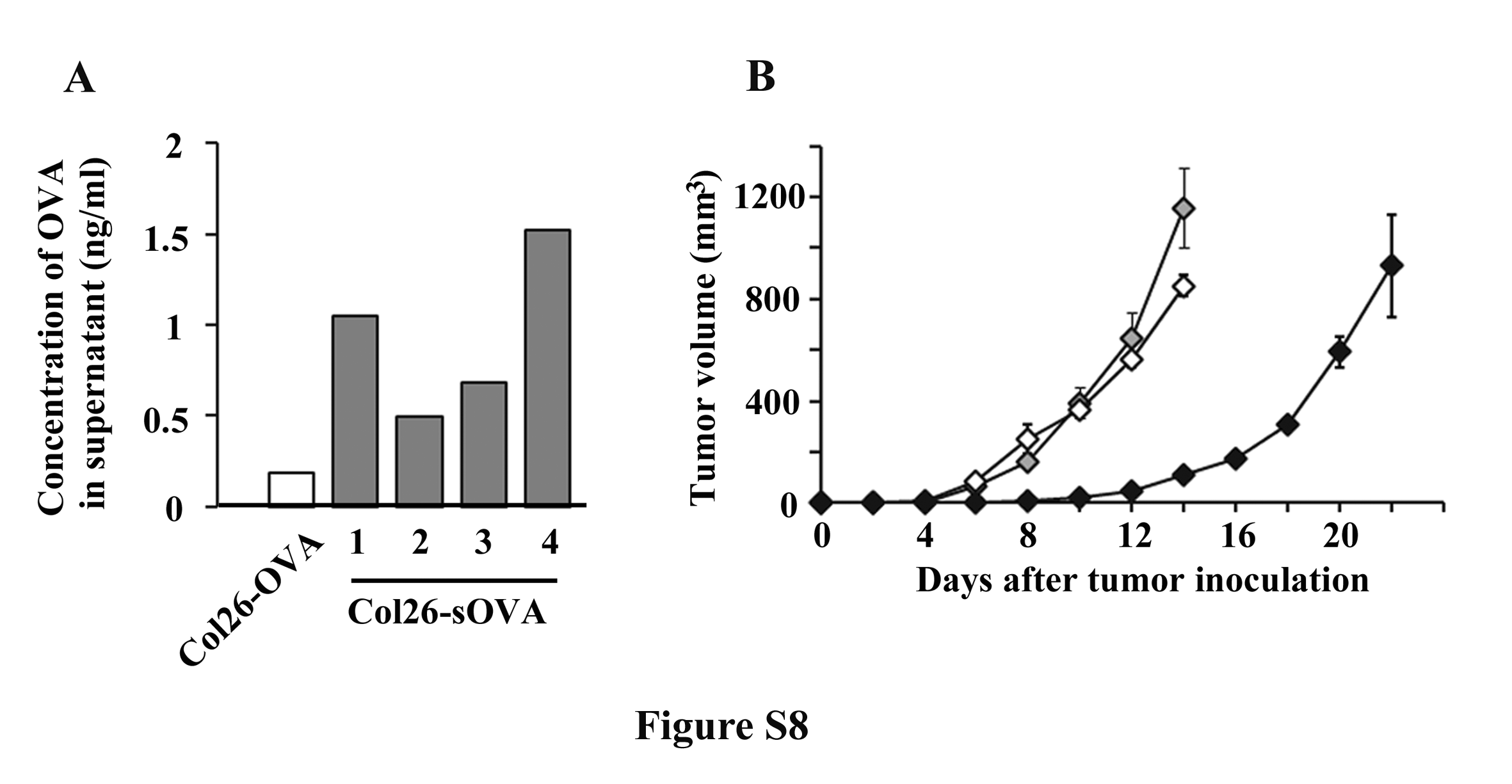

Supplement: Figure S8 — Growth of Col26 secreting OVA protein was retarded in DO11.10 mice. (A) Col26-OVA and 4 clones of Col26-sOVA were established. Culture supernatant was collected after the incubation of 1×10^5^ cells in 1 ml for 2 days. Concentration of OVA protein in the supernatant was determined. (B) Tumor size was measured at every two days after subcutaneous injection of 5×10^5^ cells into DO11.10 mice. The growth of parental Col26, Col26-OVA, and Col26-sOVA cells were represented by unfilled, gray-filled, and black-filled symbols, respectively. Tumor volume (mm3) = Length x Width x Depth/2. Data represent mean ± SD from three independent experiments. (TIF) [file pone.0041154.s008.tif]
